# Antibiotic Treatment of Pulmonary Infections: An Umbrella Review and Evidence Map

**DOI:** 10.3389/fphar.2021.680178

**Published:** 2021-10-19

**Authors:** Man Wu, Xue Yang, Jinhui Tian, Hong Fan, Yonggang Zhang

**Affiliations:** ^1^ Department of Respiratory and Critical Care Medicine, West China Hospital/West China School of Medicine, Sichuan University, Chengdu, China; ^2^ Department of Respiratory and Critical Care Medicine, Shenzhen People’s Hospital, Shenzhen, China; ^3^ Evidence-Based Medicine Center, School of Basic Medical Sciences, Lanzhou University, Lanzhou, China; ^4^ Department of Periodical Press, West China Hospital, Sichuan University, Chengdu, China

**Keywords:** pulmonary infections, antimicrobial therapy strategies, randomized controlled trials, umbrella review, evidence map

## Abstract

**Background:** Considering the global burden of pulmonary infections, there is an urgent need for optimal empirical antimicrobial therapy strategies for pulmonary infections, which should rely on reliable evidence. Therefore, we aim to investigate the optimal treatment options for pulmonary infections in adults and assess the strength of that evidence.

**Methods:** We searched PubMed, Embase, the Cochrane Library, and China Biology Medicine disc to identify systematic reviews and meta-analyses of randomized controlled trials (RCTs) focusing on antimicrobial treatments for pulmonary infections. The outcomes of the included meta-analyses should include all-cause mortality or clinical treatment success. For each meta-analysis, we estimated relative risk (RR) with 95% CI. We also created an evidence map to show the efficacy of each antimicrobial treatment strategy and the certainty of the evidence.

**Results:** Twenty-six meta-analyses and two new RCTs were included that contained 31 types of antimicrobial therapy strategies. We found that carbapenems were related to lower mortality than other β-lactams or fluoroquinolones alone or in combination with aminoglycosides for HAP patients (RR 0.76, 95% CI: 0.58–0.99). There was no statistical difference in all-cause mortality between the other antimicrobial therapy strategies. As for clinical cure, treatment with fluoroquinolones was associated with better success versus macrolides or β-lactams alone for CAP patients in both the intention-to-treat (ITT) population (RR 1.22, 95% CI: 1.02–1.47) and clinically evaluable (CE) population (RR 1.37, 95% CI: 1.11–1.68). Treatment with carbapenems showed a better clinical cure over non-carbapenems for VAP patients (RR 1.21, 95% CI: 1.05–1.4). Adjunctive inhaled antibiotics compared with intravenous antibiotics alone showed a benefit for VAP (RR 1.2, 95% CI: 1.05–1.35). In addition, adjunctive nebulized aminoglycoside for nosocomial pneumonia was associated with a higher cure rate versus intravenous antibiotics alone in the ITT population (RR 1.28, 95% CI: 1.04–1.57), while no statistical difference in clinical cure was observed between other intervention groups.

**Conclusions:** We cannot evaluate which antibiotic is the best choice for the treatment of pulmonary infection. Carbapenems or adjunctive inhaled antibiotics showed a reasonable choice for HAP or VAP. However, we do not find a statistical difference between most antimicrobial therapy strategies for CAP patients.

## Introduction

Pulmonary infections are the biggest cause of human disease burden (Cookson et al., 2017). Despite advances in antimicrobial agents, pneumonia is still the leading cause of death due to infectious diseases (Ramirez et al., 2017; Franquet et al., 2019). Epidemiologically, pneumonia can be classified into community-acquired pneumonia (CAP), hospital-acquired pneumonia (HAP), and ventilator-associated pneumonia (VAP) (Kalil et al., 2016; Burnham and Kollef, 2017).

According to our pilot search, the antimicrobial agents for the treatment of pulmonary infections could be mainly classified as follows: β-lactams (including carbapenems), fluoroquinolones, macrolides, tigecycline, aminoglycosides, linezolid, lincosamides, glycopeptides, colistin, and antifungal antibiotics (Roberts and Lipman, 2009). Patients with pneumonia are often treated with empirical antibiotics before a microbial diagnosis (Lim et al., 2009). Furthermore, the recommended empiric antibiotic therapy for pneumonia is different in the guidelines of different countries (Woodhead et al., 2011; Kalil et al., 2016; Cao et al., 2017; Torres et al., 2017). However, the quality of the available evidence has many limitations, and there is no consensus on which treatment strategy is the best one. Despite these recommendations, the abuse of antibiotics is widespread, and this clinical environment plays an important role in driving antimicrobial resistance (Cookson et al., 2017). So, researchers have performed randomized controlled trials (RCTs) and meta-analyses to find the best antimicrobial therapy strategy that effectively eliminates the infection, minimizes the risk of drug resistance, and not compromises patient safety. Vardakas et al. published a meta-analysis of RCTs and found that respiratory fluoroquinolones were associated with a higher success rate of treatment than macrolides and β-lactams for adult severe CAP but not with mortality (Vardakas et al., 2008). More recently, the meta-analysis conducted by Liu et al. showed that respiratory quinolone had the similar effectiveness and mortality compared with β-lactam with or without macrolide for non-intensive care unit (ICU)–hospitalized CAP patients (Liu et al., 2019). Systematic reviews performed by Arthur et al. did not show a difference between monotherapy and combination antibiotic regimens for VAP (Arthur et al., 2016). With the emergence of more and more systematic reviews and trials for the antimicrobial therapy for pulmonary infections, the next step is to provide and summarize the best evidence for pulmonary infection treatment to decision-makers.

The evidence mapping method has been introduced as a tool intended to complement the conventional systematic review and meta-analysis and is suitable for this issue. Thus, we performed a review and generated an evidence map to investigate the efficacy of different antimicrobial therapy strategies for pulmonary infections.

## Methods

### Search Strategy and Process of Study Selection

Two authors (Man Wu and Xue Yang) independently searched PubMed, Embase, the Cochrane Library, and China Biology Medicine disc (CBM) to investigate the antimicrobial therapy of pulmonary infections from database inception until October 1, 2020 (Supplementary Table S1). The terms and keywords used in the search included (“pulmonary infections” or “respiratory tract infections,” “community-acquired pneumonia,” “hospital-acquired pneumonia,” “ventilator-associated pneumonia,” or “pneumonia”), (“antibiotics,” “anti-bacterial agents,” or “antimicrobial”), and (“meta-analysis” or “systematic review”). The references listed in the resulting articles were also searched to identify additional relevant articles.

### Inclusion and Exclusion Criteria

The following were the inclusion criteria in our study: 1) meta-analyses or systematic reviews of RCTs focused on the efficacy of different antimicrobial therapy strategies of pulmonary infections in adults; 2) at least one of the reported outcomes was all-cause mortality (treatment or follow-up period) or clinical treatment success (clinical treatment success was assessed by test of cure in the following intention-to-treat (ITT) populations, the modified ITT (MITT) population, or clinically evaluable population); and 3) published in English or Chinese. The following exclusion criteria were applied: 1) meta-analyses or systematic reviews of observational studies; 2) previous studies were repeated; 3) they included patients who were not only with pulmonary infections but also who did not report outcomes separately for pulmonary infections. In case of multiple meta-analyses of the same intervention and results, we tended to use the largest and most recent meta-analyses. In addition, the competitive meta-analysis was screened to find additional trials that were not included in the selected meta-analysis.

### Data Extraction

Two authors (Man Wu and Xue Yang) independently extracted data from all eligible publications. The third author (Jinhui Tian) would review the data extraction and resolve conflicts. The following information was extracted from all eligible studies: first author’s name, year of publication, journal, interventions (antibiotic regimens), comparisons, number of trials, type of pulmonary infections, study search and selection criteria, outcomes of interest (all-cause mortality or clinical treatment success), method of pooling estimates (fixed or random effects), detecting publication bias, and quality assessment.

### Quality Assessment

The credibility of the included meta-analyses was independently evaluated by two authors (Man Wu and Xue Yang), and any disagreements were resolved by the third author (Jinhui Tian). The quality of all included meta-analyses were assessed by using AMSTAR 2 tool (Shea et al., 2017), which contained 16 items. The answers for each item are “yes,” “partial yes,” and “no.”

### Statistical Analysis

All the calculations were analyzed by STATA 12.0. Estimates were pooled according to Mantel–Haenszel random-effects model. The risk ratio (RR) with 95% confidence interval (95% CI) was applied to assess the effectiveness of antibiotics for treating pulmonary infections. The heterogeneity was measured by the chi‐square test and *I* ^2^ statistics test. If *I* ^2^ was less than 50%, the degree of between-study heterogeneity was considered low. If a meta-analysis included at least 10 studies, Egger’s tests were used to evaluate the publication bias (Sterne et al., 2000). We also created an evidence map showing the efficacy of antimicrobial therapy strategies and the certainty of the evidence (Farah et al., 2016). In this umbrella review, we used the Grades of Recommendation, Assessment, Development, and Evaluation (GRADE) system to assess the quality of evidence, and the system classifies quality of evidence into high, moderate, low, or very low (Supplementary Table S4) (Brozek et al., 2010).

## Results

**FIGURE 1:**
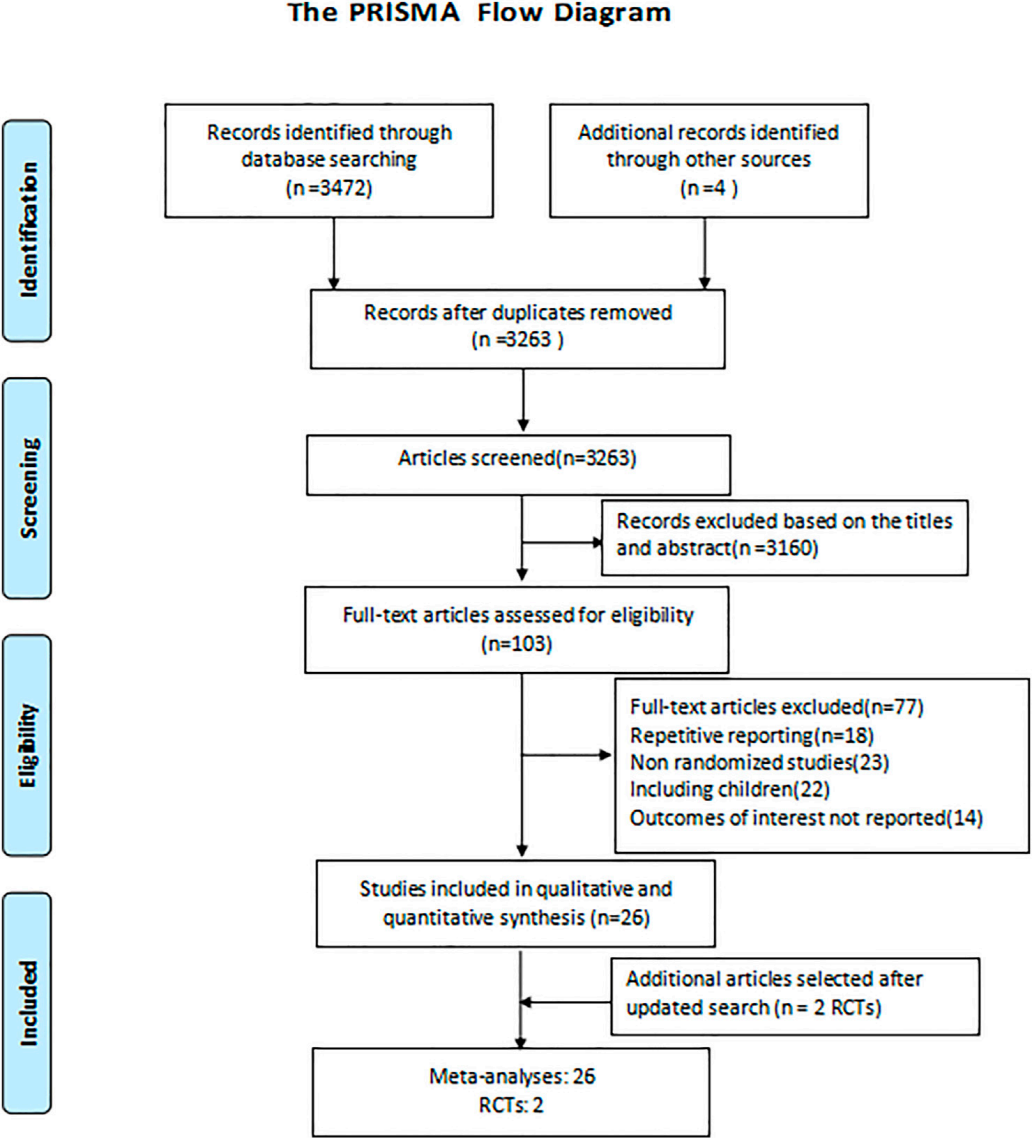
Flow diagram of the selection process.

The detailed screening and selection process is showed in the flow diagram of Figure 1. We identified a total of 3,476 citations from databases. After removing duplicates and screening all the titles and abstracts, 103 articles were identified for full-text review. We subsequently excluded 77 articles for the following reasons: repetitive reporting, non-randomized studies, including children, or outcomes of interest not reported. Ultimately, we included 26 meta-analyses and two new RCTs (Salkind et al., 2002; Mills et al., 2005; Shorr et al., 2005; Siempos et al., 2007; Vardakas et al., 2008; Liu et al., 2010; Cai et al., 2011; Eliakim-Raz et al., 2012; Lei et al., 2012; Yuan et al., 2012; Jiang et al., 2013; Skalsky et al., 2013; Garin et al., 2014; Pakhale et al., 2014; Qu et al., 2015; Raz-Pasteur et al., 2015; Shen et al., 2015; Arthur et al., 2016; Horita et al., 2016; Kalil et al., 2016; O’Donnell et al., 2018; Lan et al., 2019; Liu et al., 2019; Rui et al., 2019; Sweeney and Kalil, 2019; Wen et al., 2019; Zhang et al., 2019; Chen et al., 2020).The interventions evaluated in the meta-analyses included 31 types compared of antimicrobial therapy strategies: respiratory fluoroquinolones alone, macrolides alone, β-lactams alone, macrolides+β-lactams, respiratory fluoroquinolones+β-lactams, atypical antibiotic coverage, without atypical antibiotic coverage, tigecycline, sitafloxacin, vancomycin, linezolid, teicoplanin, carbapenems, doripenem, and adjunctive nebulized antibiotics. For the included study population, 14 meta-analyses focus on CAP, while as for nosocomial pneumonia, HAP, VAP, and pneumonia, there were 6, 1, 3, and 1 meta-analyses, respectively. Detailed characteristics of the included studies are summarized in the appendix (Supplementary Tables S2, S3).

### Quality Assessment

All the included meta-analyses were trial-level. We used the 16-item AMSTAR 2 tool to assess the methodological quality of the included articles; 46.2% of meta-analyses were judged to be “critically low/low” quality, 42.3% to be “moderate” quality, and only 11.5% to be “high” quality. The overall quality of AMSTAR 2 for each published meta-analyses is shown in Supplementary Table S1. The main flaws were lack of protocol registration, no list of excluded studies, and no publication risk assessment.

### All-Cause Mortality

**FIGURE 2:**
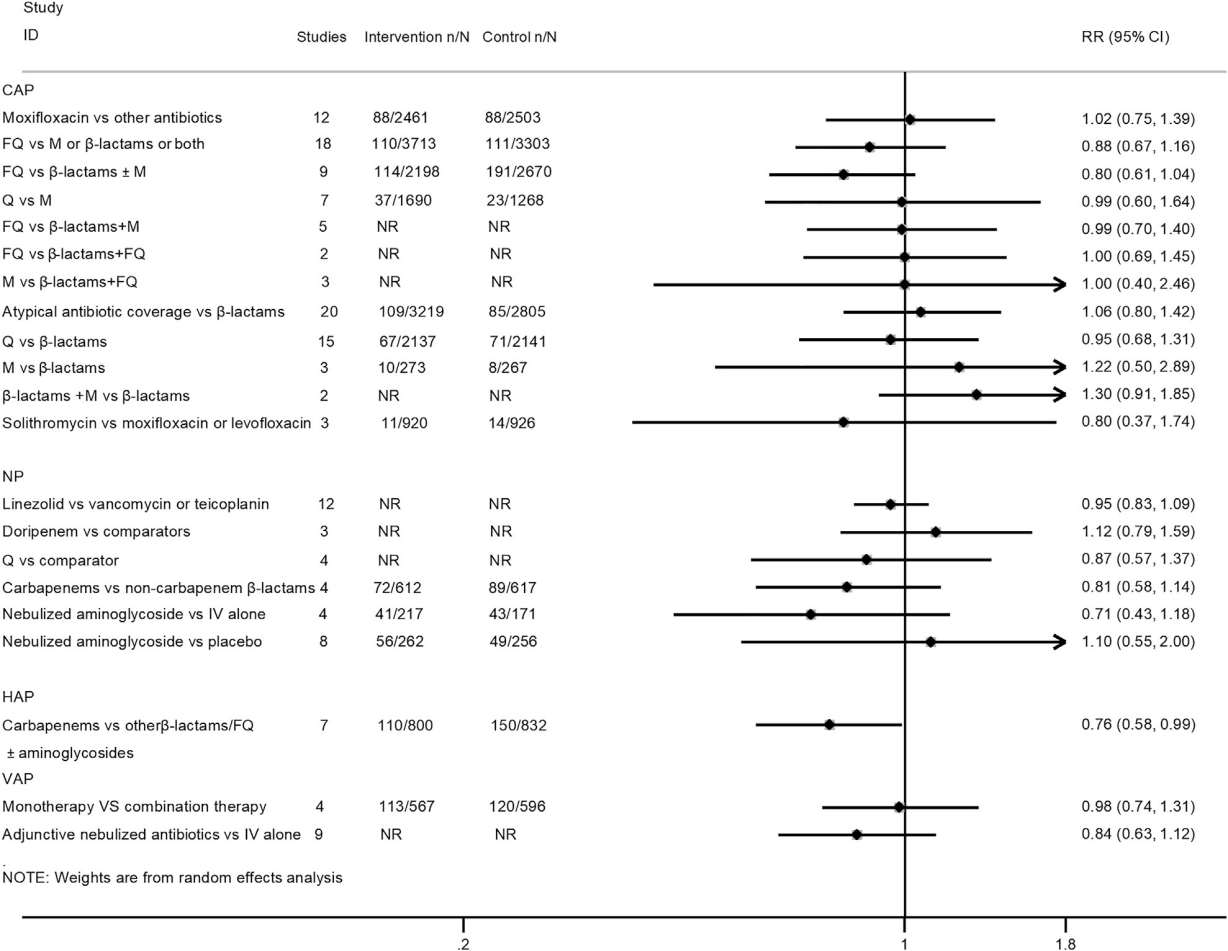
Effects of different antimicrobial therapy strategies on all-cause mortality.

Twenty-one interventions assessed the risk for all-cause mortality (Mills et al., 2005; Shorr et al., 2005; Siempos et al., 2007; Vardakas et al., 2008; Cai et al., 2011; Eliakim-Raz et al., 2012; Lei et al., 2012; Yuan et al., 2012; Jiang et al., 2013; Skalsky et al., 2013; Qu et al., 2015; Raz-Pasteur et al., 2015; Arthur et al., 2016; Horita et al., 2016; Kalil et al., 2016; O’Donnell et al., 2018; Liu et al., 2019; Rui et al., 2019; Sweeney and Kalil, 2019; Wen et al., 2019). Only the treatment with carbapenems was related to lower mortality than β-lactams or fluoroquinolones alone or in combination with aminoglycosides for HAP patients (RR 0.76, 95% CI: 0.58–0.99; very low certainty). There was no statistical difference between the other compared antimicrobial therapy strategies regarding all-cause mortality. The pooled estimates for the outcomes are presented in Figure 2.

### Clinical Treatment Success

**FIGURE 3:**
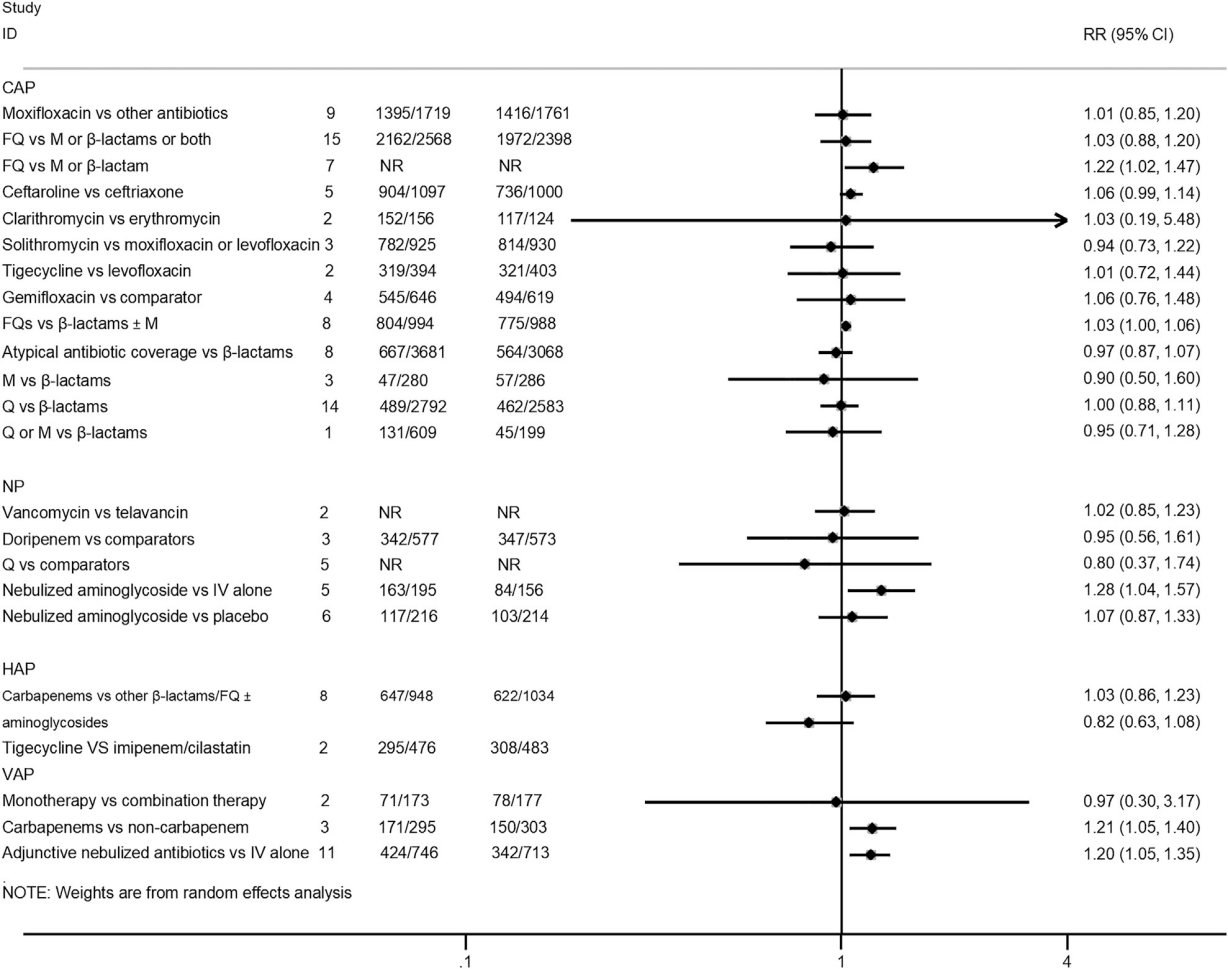
Clinical treatment success analysis based on intention-to-treat population.

**FIGURE 4:**
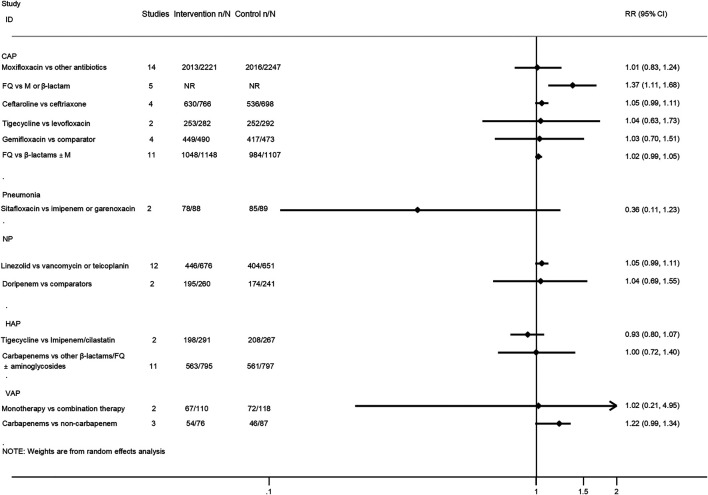
Clinical treatment success analysis based on clinically evaluable population.

Twenty-five interventions assessed the clinical treatment success based on the ITT, MITT, or CE population (Salkind et al., 2002; Mills et al., 2005; Shorr et al., 2005; Siempos et al., 2007; Vardakas et al., 2008; Cai et al., 2011; Eliakim-Raz et al., 2012; Lei et al., 2012; Yuan et al., 2012; Jiang et al., 2013; Pakhale et al., 2014; Raz-Pasteur et al., 2015; Shen et al., 2015; Qu et al., 2015; Arthur et al., 2016; Kalil et al., 2016; O’Donnell et al., 2018; Lan et al., 2019; Liu et al., 2019; Rui et al., 2019; Sweeney and Kalil, 2019; Wen et al., 2019; Zhang et al., 2019; Chen et al., 2020). Treatment with fluoroquinolones was associated with a better success rate than macrolides or β-lactam antibiotics for CAP patients in both the ITT population (RR 1.22, 95% CI: 1.02–1.47; low certainty) and CE population (RR 1.37, 95% CI: 1.11–1.68; moderate certainty). Moreover, the treatment of CAP patients with ceftaroline had a similar cure rate when compared with ceftriaxone (MITT population, RR 1.06, 95% CI: 0.99–1.14, moderate certainty; CE population, RR 1.05, 95% CI: 0.99–1.11, moderate certainty). As for VAP patients, treatment with carbapenems showed a better clinical cure than non-carbapenem antibiotics (ITT population, RR 1.21, 95% CI: 1.05–1.4, moderate certainty), and adjunctive nebulized antibiotics comparing with intravenous antibiotics alone showed a benefit (RR 1.2, 95% CI: 1.05–1.35; high certainty). In addition, nebulized aminoglycoside for nosocomial pneumonia was associated with a higher cure rate than intravenous antibiotics alone in the ITT population (RR 1.28, 95% CI: 1.04–1.57; moderate certainty), while the efficacy of tigecycline in the treatment of HAP patients was similar compared with imipenem/cilastatin drugs (ITT population, RR 0.82, 95% CI: 0.63–1.08, low certainty; CE population, RR 0.93, 95% CI: 0.8–1.07, low certainty). No statistical difference was observed between other intervention groups regarding the clinical treatment success. The pooled estimates for the outcomes are presented in Figures 3, 4.

### Evidence Map

**FIGURE 5:**
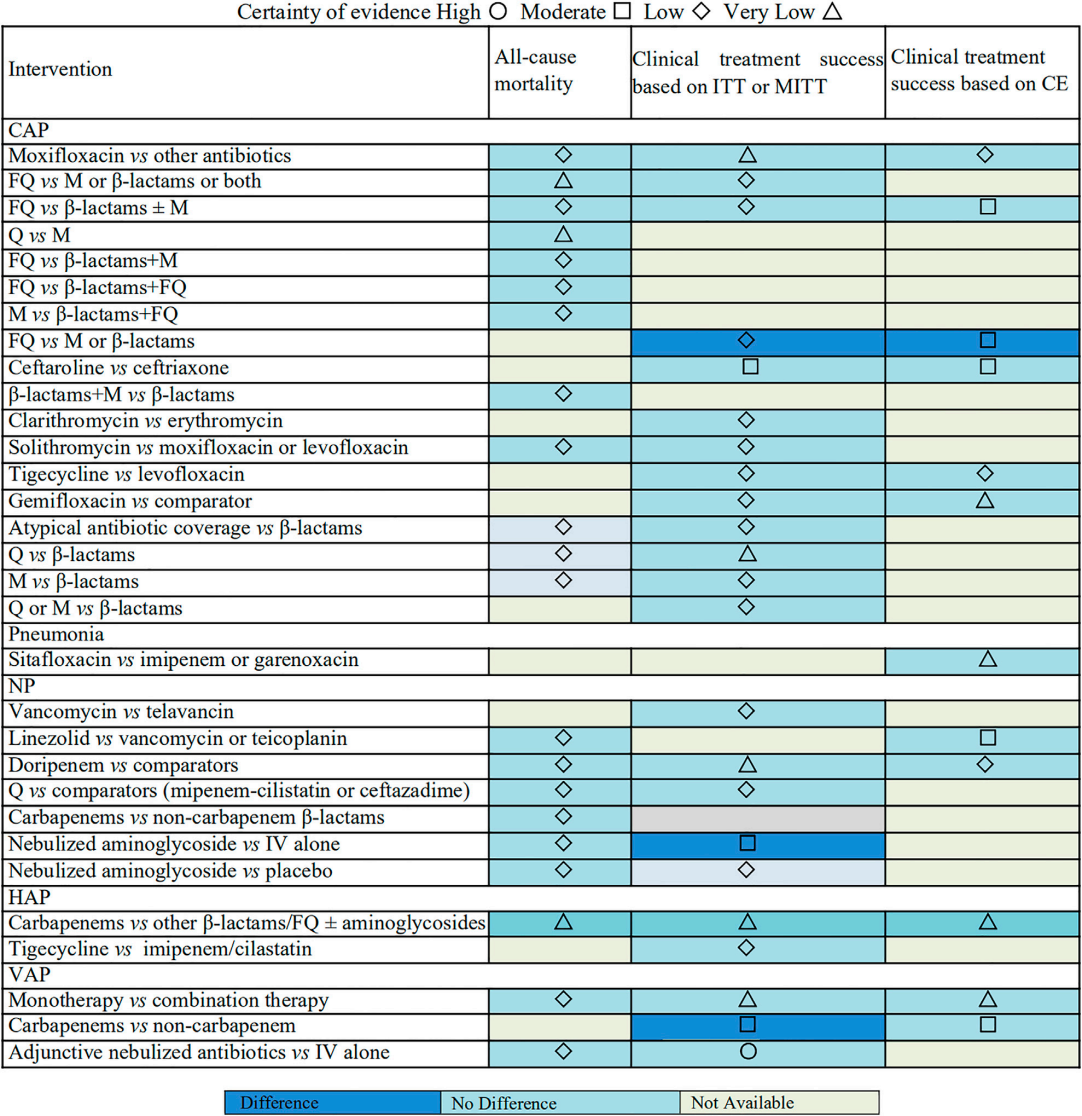
Evidence map of all-cause mortality and clinical treatment success.

An evidence map was conducted to summarize the findings for included antimicrobial therapy strategies (Figure 5). The map shows the lack of significant effects on clinical treatment success and all-cause mortality for among the most included antimicrobial therapy strategies for patients with pulmonary infections. The certainty of evidence varies from very low to low between most intervention groups and control groups. The evidence was graded as moderate for 14.55% (*n* = 8) of the associations, and only one intervention has high-quality evidence compared to the control group.

## Discussion

Our comprehensive review provides a direct quantitative comparison of various antimicrobial interventions for patients with pneumonia regarding all-cause mortality or clinical treatment success outcomes. After assessing the strength, direction, and the consistency of the associations, we found some high strength of evidence that these 2 types of antimicrobial interventions (carbapenems or adjunctive nebulized antibiotics in VAP) had a higher cure rate than the compared antibiotics. Additionally, the treatment with carbapenems was related to lower mortality than regimens in HAP patients with very low evidence. Other interventions such as comparisons of fluoroquinolones with β-lactams or macrolides or both, or β-lactams plus fluoroquinolones, β-lactams plus macrolides versus β-lactams, tigecycline versus levofloxacin, and atypical antibiotic coverage versus β-lactams in CAP and additionally, carbapenems versus other β-lactams, linezolid versus vancomycin or teicoplanin, doripenem versus other antibiotics, vancomycin versus telavancin, quinolones versus comparators (imipenem–cilastatin or ceftazidime), and nebulized aminoglycoside versus placebo in nosocomial pneumonia, tigecycline versus imipenem/cilastatin in HAP, and monotherapy versus combination therapy in VAP did not show a significant effect on all-cause mortality or clinical treatment success outcomes (with very low to moderate certainty evidence).

Fluoroquinolones, β-lactams, and macrolides are the main antibiotics that have dominated the market for many years, and they are active against the main pathogens of CAP (Suda et al., 2017). The optimal antimicrobial strategies in CAP patients have been controversial. An earlier meta-analysis conducted by Salkind et al. more than a decade ago found that oral fluoroquinolones showed modest therapeutic benefit compared with β-lactams or macrolides in CAP (Salkind et al., 2002). However, these recent meta-analysis found that there was no statistical treatment difference between fluoroquinolones and β-lactams or macrolides or both in CAP, regarding mortality or clinical treatment success outcomes (Skalsky et al., 2013; Liu et al., 2019). Furthermore, the pathogens of CAP were traditionally divided into “typical” and “atypical,” while, evidence of empirical broad coverage treatment for CAP patients is still insufficient. There was low-quality evidence that the empirical coverage of atypical pathogens (mainly quinolones or macrolides) did not show an advantage in survival or efficacy over the coverage of typical pathogens (mainly β-lactams). In summary, fluoroquinolones had similar therapeutic effects for CAP patients compared with macrolides or β-lactams or both. With the emergence of more and more antibiotics, ceftaroline as a new cephalosporin has emerged, with broad-spectrum activity against many common pathogens that cause CAP (Pfaller et al., 2018). Fortunately, there is evidence that the clinical efficacy of ceftaroline was similar compared with ceftriaxone for CAP patients based on clinical treatment success.

Selection of initial empiric appropriate antimicrobial treatment of patients with HAP or VAP significantly improves outcomes based on the risk for multidrug-resistant (MDR) pathogens (Kalil et al., 2016). As for HAP or VAP patients, some guidelines recommend monotherapy for patients with low risk of MDR bacteria and combination therapy for patients with high risk of drug resistance. Combination therapy seems to be preferred for severe infections, especially ICU patients in the clinic, while no difference was found between monotherapy and combination therapy for VAP in the Cochrane review conducted by Arthur et al. (2016). Since high risk of MDR bacteria was not identified in the included patients, these data may not be applicable to all patients. There is evidence that carbapenems as an empiric antibiotic therapy were related to a statistically significant increase in the clinical treatment success versus non-carbapenem for VAP. In addition, Siempos et al. found that carbapenems did not show a better clinical efficacy versus comparators but reduced all-cause mortality for HAP (Siempos et al., 2007). Tigecycline has been often used for treatment of many serious infectious diseases. Although tigecycline has similar clinical efficacy compared with imipenem/cilastatin based on clinical treatment success rate, it has more frequency of adverse events (Shen et al., 2015; Arthur et al., 2016). Although the small number of trials and the small scale included in those reviews may limit meaningful clinical applications, we could not evaluate which antibiotic is the best choice for the treatment of patients with HAP or VAP. However, carbapenems should be regarded as a reliable option for empirical treatment of adult patients with HAP or VAP. In the future, prospective randomized studies should be conducted to evaluate whether carbapenems are superior to more restrictive antibiotics or combination therapy. Unfortunately, in the subgroup analysis of *Pseudomonas aeruginosa*, it was found that carbapenems showed a lower effectiveness than comparators. It may be related to the high resistance rate of *Pseudomonas aeruginosa* to carbapenems (Labarca et al., 2016; Khadem et al., 2017). For patients with HAP/VAP caused by *Pseudomonas aeruginosa*, the guidelines recommend selecting antibiotics for definitive (non-empirical) treatment based on the results of antimicrobial susceptibility tests (Kalil et al., 2016).

Nebulized inhaled antibiotic is one of the methods proposed in recent years to treat resistant organisms (including MDR, extensively drug-resistant and pan-resistant organisms) (Kalil et al., 2016). There are four aerosolized antibiotics that have received approval either from European Medicines or the U.S. Food and Drug Administration: aztreonam, amikacin liposome, colistin, and tobramycin (Quon et al., 2014; FDA, 2018). The meta-analysis conducted by Sweeney et al. showed that adjunctive inhaled antibiotics (including amikacin liposome, gentamicin, colistin, and tobramycin) may benefit patients with VAP caused by MDR or difficult-to-treat organisms, especially limited intravenous antibiotic options, regarding clinical treatment success outcomes (Sweeney and Kalil, 2019), which have combined both the recent two trials (INHALE and IASIS trials). Although the disappointing results of the two recent trials, the final meta-analysis results still show a benefit of adjunctive inhaled antibiotics for patients with VAP. The fact that should not be ignored is that INHALE and IASIS enrolled patients who were not only infected with MDR organisms but also had limited intravenous antibiotic options. Another meta-analysis also suggested adjunctive inhaled aminoglycoside antibiotics, which showed better efficacy in the treatment of HAP or VAP (Rui et al., 2019). In addition, some expert groups also believe that for patients who could not respond to intravenous antibiotics alone, regardless of whether the infected organism is MDR, it is reasonable to consider adjunctive inhaled antibiotic therapy as the last treatment option (Kalil et al., 2016). Part of the reason for the clinical benefit of adjunctive inhaled antibiotic therapy is that the antibiotic efficacy against bacteria in purulent secretions may require an antibiotic concentration greater than 10–25 times the minimum inhibitory concentration (MIC), which could not be achieved by intravenous treatment alone, which however inhaled antibiotic therapy may achieve (Petitcollin et al., 2016; Boisson et al., 2017; Wong et al., 2019). However, the optimal administration, dosage, and safety of inhaled antibiotic therapy are not very clear, and more research on these aspects is needed in the future.

Nevertheless, our research has several flaws. First, several meta-analyses included fewer trials, leading to small study effects and affecting research results (Schwarzer et al., 2015), and had publication bias. Second, the included meta-analyses and RCTs had inherent limitations, such as inconsistent baseline characteristics of the included populations, publication bias, and inconsistent follow-up time. Third, because our research focused on providing broad evidence for empirical antimicrobial therapy strategies for patients with pulmonary infection from the existing meta-analyses, we could not analyze interventions based on important subgroups, such as comorbidities, etiology, severity of illness, and age. Our study’s virtues were that data were obtained only from RCTs and their meta-analyses. Additionally, our study quantitatively analyzed and compared the effects of various empirical antimicrobial therapy strategies on the mortality and clinical cure rates of patients with pulmonary infection. An evidence map for the antimicrobial treatment of pulmonary infections was not identified from any previous systematic reviews; therefore, our study would be the first to utilize an evidence map to identify evidence gaps and to facilitate evidence communication for pulmonary infections.

In summary, by comparing various empirical antimicrobial therapy strategies on the mortality and clinical efficacy of patients with pulmonary infection, we found evidence that carbapenems may show better clinical efficacy than non-carbapenems for HAP or VAP patients. Adjunctive inhaled antibiotics are a reasonable choice for HAP or VAP patients with MDR and even limited intravenous antibiotic options. For CAP patients, we did not find differences between fluoroquinolones and β-lactams or macrolides alone or both regarding mortality and clinical treatment success. Empirical coverage atypical pathogens did not show advantage in survival or efficacy compared to coverage of typical pathogens. Overall, these findings are limited by the poor quality of the evidence. This research could provide evidence for clinicians to choose empirical antimicrobial treatment strategies and guide new research.

## Abbreviations

CAP, community-acquired pneumonia; CE, clinically evaluable; CI, confidence interval; FQ, fluoroquinolones; HAP, hospital-acquired pneumonia; ICU, intensive-care unit; ITT, intention-to-treat; IV, intravenous; M, macrolides; MDR, multidrug-resistant; MITT, modified intention-to-treat; NP, nosocomial pneumonia; Q, quinolones; RCT, randomized controlled trial; RR, rate ratio; VAP, ventilator-associated pneumonia.
