# Supplementary material for: Antibiotic Treatment of Pulmonary Infections: An Umbrella Review and Evidence Map
Source: Front Pharmacol. 2021 Oct 19;12:680178. doi: 10.3389/fphar.2021.680178 (PMC8560894; doi:10.3389/fphar.2021.680178)
Supplement: Supplementary file 1 [file DataSheet1.docx]

Supplement Table 1. Electronic search strategies (PubMed)

| **Category** | **Pulmonary infections** | **Antibiotics** | **Meta-analysis** |
| --- | --- | --- | --- |
| **Search term** | #1.pulmonary infections | #8. antibiotic(s) | #12. meta-analysis |
|  | #2. respiratory tract infections | #9. antimicrobial | #13. systematic review |
|  | #3. pneumonia | #10. anti-bacterial agents |  |
|  | #4. community-acquired pneumonia |  |  |
|  | #5. hospital-acquired pneumonia |  |  |
|  | #6. ventilator-associated pneumonia |  |  |
| **Small sum** | #7. (or/1-6) | #11. (or/8-9) | #14. (12 or 13) |
| **Over sum** | #15. (7 and 11 and 14) | | |

Supplement Table 2. Characteristics and quality assessment of the included meta-analysis

| **Study** | **Intervention** | **Studies** | **Patients** | **Guideline** | **Quality** | **Publication**  **bias** | **Type of data** | **Method of pooling estimates** | **Quality**  **(AMSTAR)** |
| --- | --- | --- | --- | --- | --- | --- | --- | --- | --- |
| Yuan X 2012^[[1](#_ENREF_1)]^ | Moxifloxacin vs other antibiotics | 14 | CAP | NR | Jadad | Funnel plot and Eggers test | Trial level | Fixed effects or random effects | Moderate |
| Vardakas KZ 2008^[[2](#_ENREF_2)]^ | Respiratory fluoroquinolones vs macrolides or β-lactams or both | 23 | CAP | NR | Jadad | Funnel plot and Eggers test | Trial level | Fixed effects or random effects | Moderate |
| Liu ST 2019^[[3](#_ENREF_3)]^ | Respiratory fluoroquinolones vs β-lactams ± macrolides | 22 | CAP (inpatient) | NR | Cochrane | Funnel plot and Eggers test | Trial level | Fixed effects or random effects | Moderate |
| Skalsky K 2013^[[4](#_ENREF_4)]^ | Quinolones vs macrolides | 7 | CAP(inpatients or outpatients) | NR | Cochrane | NR | Trial level | Fixed effects | Low |
| Raz-Pasteur A 2015^[[5](#_ENREF_5)]^ | Respiratory fluoroquinolones or macrolides alone vs combined with β-lactams | 16 | CAP(ICU/non-ICU/outpatients) | NR | Cochrane | NR | Trial level | Fixed effects | Low |
| Salkind AR 2002^[[6](#_ENREF_6)]^ | Fluoroquinolones vs macrolides or β-lactams | 12 | CAP | NR | NR | NR | Trial level | Fixed effects or random effects | Critically low |
| Eliakim-Raz N 2012^[[7](#_ENREF_7)]^ | Atypical vs without atypical antibiotic coverage (β-lactams ) | 28 | CAP(inpatients) | NR | Cochrane | Funnel plot | Trial level | Fixed effects or random effects | Moderate |
| Mills GD 2005^[[8](#_ENREF_8)]^ | Atypical antibiotic coverage vs β-lactams | 18 | Non-severe CAP | NR | NR | NR | Trial level | Fixed effects or random effects | Critically low |
| Lan SH 2019^[[9](#_ENREF_9)]^ | Ceftaroline vs ceftriaxone | 5 | CAP | NR | Cochrane | NR | Trial level | Fixed effects or random effects | Low |
| Horita N 2016^[[10](#_ENREF_10)]^ | β-lactams + macrolides vs β-lactams | 2 | CAP | PRISMA | Cochrane | Funnel plot and Begg | Trial level | Random effects | Moderate |
| Pakhale S 2014^[[11](#_ENREF_11)]^ | Clarithromycin vs erythromycin | 2 | CAP(outpatients) | NR | Cochrane | Funnel plot | Trial level | Fixed effects | High |
| Wen JL 2019^[[12](#_ENREF_12)]^ | Solithromycin vs moxifloxacin or levofloxacin | 3 | CAP | NR | Jadad | Funnel plot | Trial level | Fixed effects or random effects | Moderate |
| Cai Y 2011^[[13](#_ENREF_13)]^ | Tigecycline vs levofloxacin | 2 | CAP | NR | Jadad | Funnel plot | Trial level | Fixed effects or random effects | Moderate |
| Zhang L 2012^[[14](#_ENREF_14)]^ | Gemifloxacin vs comparator (amoxicillin/clavulanate /levofloxacin/ceftriaxone+macrolide/trovafloxacin) | 4 | CAP | NR | Jadad | Funnel plot and Eggers test | Trial level | Fixed effects or random effects | Moderate |
| Shen FC 2015^[[15](#_ENREF_15)]^ | Tigecycline vs imipenem/cilastatin | 2 | HAP | PRISMA | Cochrane | Funnel plot and Eggers test | Trial level | Fixed effects or random effects | High |
| Chen CK 2020^[[16](#_ENREF_16)]^ | Sitafloxacin vs imipenem or garenoxacin | 2 | pneumonia | NR | Cochrane | NR | Trial level | Random effects | Critically low |
| Zhang Y 2019^[[17](#_ENREF_17)]^ | Vancomycin vs telavancin | 2 | NP | PRISMA | Cochrane | NR | Trial level | Random effects | Low |
| Jiang H 2013^[[18](#_ENREF_18)]^ | Linezolid vs vancomycin or teicoplanin | 12 | NP | NR | Jadad | Eggers test and Begg | Trial level | Fixed effects or random effects | Moderate |
| Qu XY 2015^[[19](#_ENREF_19)]^ | Doripenem vs comparators | 3 | NP | NR | Jadad | NR | Trial level | Fixed effects or random effects | Critically low |
| Shorr AF 2005^[[20](#_ENREF_20)]^ | Quinolones vs comparators (mipenem-cilistatin or ceftazadime) | 5 | NP | NR | Jadad | Begg | Trial level | Fixed effects | Low |
| O’Donnell JN 2018^[[21](#_ENREF_21" \o "Nicholas O"Donnell, 2018 #3445)]^ | Carbapenems vs non-carbapenem β-lactams | 4 | NP | NR | Cochrane | Funnel plot | Trial level | Fixed effects or random effects | Moderate |
| Siempos II 2007^[[22](#_ENREF_22)]^ | Carbapenems vs other β-lactams or fluoro- quinolones or in combination with aminoglycosides | 12 | HAP | NR | Jadad | Funnel plot and Eggers test | Trial level | Random effects | Moderate |
| Arthur LE 2016^[[23](#_ENREF_23)]^ | Monotherapy vs combination therapy  carbapenems vs non-carbapenems | 4/3 | VAP | NR | Cochrane | Funnel plot | Trial level | Fixed effects | High |
| Sweeney DA 2019^[[24](#_ENREF_24)]^ | Adjunctive nebulized antibiotics vs IV alone | 10 | VAP | NR | NR | Eggers test | Trial level | random effects | Low |
| Andre C. Kalil 2016 ^[[25](#_ENREF_25)]^ | Adjunctive nebulized antibiotics vs IV alone | 9 | VAP | NR | NR | Eggers test | Trial level | random effects | Low |
| Li R 2019^[[26](#_ENREF_26)]^ | Nebulized antibiotics vs IV alone+placebo | 12 | NP | NR | Jadad | Eggers test and Begg | Trial level | Fixed effects or random effects | Low |

CAP: community-acquired pneumonia; HAP：hospital-acquired pneumonia; VAP: ventilator-associated pneumonia; NP: nosocomial pneumonia; IV: Intravenous injection; NR: not report.

Supplement Table 3. Baseline characteristics of randomized controlled trials included in analysis

| **Study** | **Patients** | **Intervention** | **Number of patients** | **Outcomes RR (95%CI)** |
| --- | --- | --- | --- | --- |
| Lu Q 2011^[[27](#_ENREF_27)]^ | VAP | Nebulized ceftazidime or amikacin vs IV alone | 40 | Clinical cure: RR 1.27(0.78-2.08); Mortality: RR 2(0.2-20.33) |
| Grain 2014^[[28](#_ENREF_28)]^ | CAP | Cefuroxime or amoxicillin/clavulanate vs clarithromycin | 580 | Mortality: RR 0.719(0.325-1.593) |

CAP: community-acquired pneumonia; VAP；ventilator-associated pneumonia; NP: nosocomial pneumonia; IV: Intravenous injection; RR: risk ratios.

Supplement Table 4. GRADE scale for quality assessment of evidence

1. Certainty assessment of mortality

| Certainty assessment | | | | | | | | Effect | Certainty |
| --- | --- | --- | --- | --- | --- | --- | --- | --- | --- |
| Interventions vs comparisons | Studies | Study design | Risk of bias | Inconsistency | Indirectness | Imprecision | Publication bias | Relative  (95%CI) |  |
| Mortality for CAP | | | | | | | | | |
| Moxifloxacin vs other antibiotics | 12 | RCT | not serious | not serious | not serious | very serious | not serious | RR 1.02(0.75-1.39) | low |
| FQ vs M or β-lactams or both | 18 | RCT | serious | not serious | not serious | very serious | not serious | RR 0.88(0.67-1.16) | very low |
| FQ vs β-lactams ± M | 9 | RCT | serious | not serious | not serious | serious | not serious | RR 0.8(0.61-1.04) | low |
| Q vs M or β-lactams | 7 | RCT | serious | not serious | not serious | very serious | not serious | RR 0.99(0.6-1.64) | very low |
| FQ vs β-lactams+M | 5 | RCT | not serious | not serious | not serious | very serious | not serious | RR 0.99(0.7-1.4) | low |
| FQ vs β-lactams+FQ | 2 | RCT | not serious | not serious | not serious | very serious | not serious | RR 1(0.69-1.45) | low |
| M VS β-lactams+FQ | 3 | RCT | not serious | not serious | not serious | very serious | not serious | RR 1(0.4-2.46) | low |
| β-lactams +M vs β-lactams | 2 | RCT | not serious | not serious | not serious | very serious | not serious | RR 1.3(0.91-1.83) | low |
| Solithromycin vs moxifloxacin or levofloxacin | 3 | RCT | not serious | not serious | not serious | very serious | not serious | RR 0.8(0.37-1.74) | low |
| Atypical antibiotic coverage vs β-lactams | 20 | RCT | not serious | not serious | not serious | very serious | not serious | RR 1.06(0.8-1.42) | low |
| Q vs β-lactams | 15 | RCT | not serious | not serious | not serious | very serious | not serious | RR 0.95(0.68-1.31) | low |
| M vs β-lactams | 3 | RCT | not serious | not serious | not serious | very serious | not serious | RR 1.22(0.5-2.89) | low |
| Mortality for NP | | | | | | | | | |
| Linezolid vs vancomycin or teicoplanin | 12 | RCT | not serious | not serious | not serious | very serious | not serious | RR 0.95(0.83-1.09) | low |
| Doripenem vs comparators | 3 | RCT | not serious | not serious | not serious | very serious | not serious | RR 1.12(0.79-1.59) | low |
| Q vs comparators (mipenem -cilistatin or Ceftazadime) | 4 | RCT | not serious | not serious | not serious | very serious | not serious | RR 0.87(0.57-1.37) | low |
| Carbapenems vs non-carbapenem β-lactams | 4 | RCT | not serious | not serious | not serious | very serious | not serious | RR 0.81(0.58-1.14) | low |
| Nebulized aminoglycoside vs IV alone | 4 | RCT | not serious | not serious | not serious | very serious | not serious | RR 0.71(0.43-1.18) | low |
| Nebulized aminoglycoside vs placebo | 8 | RCT | not serious | not serious | not serious | very serious | not serious | RR 1.1(0.55-2) | low |
| Mortality for HAP | | | | | | | | | |
| Carbapenems vs other β-lactams/FQ ± aminoglycosides | 7 | RCT | serious | serious | not serious | serious | not serious | RR 0.76(0.58-0.99) | very low |
| Mortality for VAP | | | | | | | | | |
| Monotherapy vs combination therapy | 4 | RCT | not serious | not serious | not serious | very serious | not serious | RR 0.98(0.74-1.31) | low |
| Adjunctive nebulized antibiotics vs IV alone | 9 | RCT | not serious | not serious | not serious | very serious | not serious | RR 0.84(0.63-1.12) | low |

CAP: community-acquired pneumonia; HAP：hospital-acquired pneumonia; VAP: ventilator-associated pneumonia; NP: nosocomial pneumonia; RCT: randomized controlled trials; RR: risk ratios; FQ: fluoroquinolones; M: macrolides; Q: quinolones; IV: Intravenous injection.

B.Certainty assessment of clinical treatment success based on ITT or MITT

| Certainty assessment | | | | | | | | Effect | Certainty |
| --- | --- | --- | --- | --- | --- | --- | --- | --- | --- |
| Interventions vs comparisons | Studies | Study design | Risk of bias | Inconsistency | Indirectness | Imprecision | Publication bias | Relative  (95%CI) |  |
| Clinical treatment success based on ITT or MITT for CAP | | | | | | | | | |
| Moxifloxacin vs other antibiotics | 9 | RCT | serious | not serious | not serious | very serious | not serious | RR 1.01(0.85-1.20) | very low |
| FQ vs M or β-lactams or both | 15 | RCT | serious | not serious | not serious | very serious | not serious | RR 1.03(0.88-1.20) | very low |
| FQ vs M or β-lactam | 7 | RCT | serious | not serious | not serious | serious | not serious | RR 1.22(1.02-1.47) | low |
| Ceftaroline vs ceftriaxone | 5 | RCT | not serious | not serious | not serious | serious | not serious | RR 1.06(0.99-1.14) | moderate |
| Clarithromycin vs erythromycin | 2 | RCT | not serious | not serious | not serious | very serious | not serious | RR 1.03(0.19-5.48) | low |
| Solithromycin vs moxifloxacin or levofloxacin | 3 | RCT | not serious | not serious | not serious | very serious | not serious | RR 0.94(0.73-1.22) | low |
| Tigecycline vs levofloxacin | 2 | RCT | not serious | not serious | not serious | very serious | not serious | RR 1.01(0.72-1.44) | low |
| Gemifloxacin vs comparator | 4 | RCT | not serious | not serious | not serious | very serious | not serious | RR 1.06(0.76-1.48) | low |
| FQs vs β-lactams ± M | 8 | RCT | serious | not serious | not serious | serious | not serious | RR 1.03(1-1.06) | low |
| Atypical antibiotic coverage vs β-lactams | 18 | RCT | not serious | not serious | not serious | very serious | not serious | RR 0.97(0.87-1.07) | low |
| M vs β-lactams | 3 | RCT | not serious | not serious | not serious | very serious | not serious | RR 0.9(0.5-1.6) | low |
| Q vs β-lactams | 14 | RCT | not serious | not serious | not serious | very serious | serious | RR 1(0.88-1.11) | very low |
| Q or M vs β-lactams | 1 | RCT | not serious | not serious | not serious | very serious | not serious | RR 0.95(0.71-1.28) | low |
| Clinical treatment success based on ITT or MITT for NP | | | | | | | | | |
| Vancomycin VS Telavancin | 2 | RCT | not serious | not serious | not serious | very serious | not serious | RR 1.02(0.85-1.23) | low |
| Doripenem vs comparators | 3 | RCT | not serious | serious | not serious | very serious | not serious | RR 0.95(0.56-1.61) | very low |
| Q vs comparators | 5 | RCT | not serious | not serious | not serious | very serious | not serious | RR 0.8(0.37-1.74) | low |
| Nebulized aminoglycoside vs IV alone | 5 | RCT | serious | not serious | not serious | serious | not serious | RR 1.28(1.04-1.57) | moderate |
| Nebulized aminoglycoside vs placebo | 6 | RCT | not serious | not serious | not serious | very serious | not serious | RR 1.07(0.87-1.33) | low |
| Clinical treatment success based on ITT or MITT for HAP | | | | | | | | | |
| Tigecycline vs imipenem /cilastatin | 2 | RCT | not serious | not serious | not serious | very serious | not serious | RR 0.82(0.63-1.08) | low |
| Carbapenems vs other β-lactams/FQ ± aminoglycosides | 8 | RCT | serious | not serious | not serious | very serious | not serious | RR 1.03(0.86-1.23) | very low |
| Clinical treatment success based on ITT or MITT for VAP | | | | | | | | | |
| Monotherapy vs combination therapy | 2 | RCT | not serious | serious | not serious | very serious | not serious | OR 0.97(0.3-3.17) | very low |
| Carbapenems vs non-carbapenems | 3 | RCT | serious | not serious | not serious | not serious | not serious | RR 1.21(1.05-1.4) | moderate |
| Adjunctive nebulized antibiotics vs IV alone | 11 | RCT | not serious | not serious | not serious | not serious | not serious | RR 1.2(1.05-1.35) | high |

CAP: community-acquired pneumonia; HAP：hospital-acquired pneumonia; VAP: ventilator-associated pneumonia; NP: nosocomial pneumonia; RCT: randomized controlled trials; MITT: modified intention-to-treat; ITT: intention-to-treat; RR: risk ratios; FQ: fluoroquinolones; M: macrolide; Q: quinolones; IV: Intravenous injection.

C. Certainty assessment of Clinical treatment success based on CE

| Certainty assessment | | | | | | | | Effect | Certainty |
| --- | --- | --- | --- | --- | --- | --- | --- | --- | --- |
| Interventions VS comparisons | Studies | Study design | Risk of bias | Inconsistency | Indirectness | Imprecision | Publication bias | Relative  (95%CI) |  |
| Clinical treatment success based on CE for CAP | | | | | | | | | |
| Moxifloxacin vs other antibiotics | 14 | RCT | not serious | not serious | not serious | very serious | not serious | RR 1.01(0.83-1.24) | low |
| FQ vs M or β-lactam | 5 | RCT | serious | not serious | not serious | not serious | not serious | RR 1.37(1.11-1.68) | moderate |
| Ceftaroline vs ceftriaxone | 4 | RCT | not serious | not serious | not serious | serious | not serious | RR 1.05(0.99-1.11) | moderate |
| Tigecycline vs levofloxacin | 2 | RCT | not serious | not serious | not serious | very serious | not serious | RR 1.04(0.63-1.73) | low |
| Gemifloxacin vs comparator | 4 | RCT | serious | not serious | not serious | very serious | not serious | RR 1.03(0.7-1.51) | very low |
| FQs vs β-lactams ± M | 11 | RCT | serious | not serious | not serious | not serious | not serious | RR 1.02(0.99-1.05) | moderate |
| Clinical treatment success based on CE for Pneumonia | | | | | | | | | |
| Sitafloxacin vs imipenem or garenoxacin | 2 | RCT | serious | not serious | not serious | very serious | not serious | RR 0.36(0.11-1.23) | very low |
| Clinical treatment success based on CE for NP | | | | | | | | | |
| Doripenem vs comparators | 2 | RCT | not serious | not serious | not serious | very serious | not serious | RR 1.04(0.69-1.55) | low |
| Linezolid vs vancomycin or teicoplanin | 12 | RCT | not serious | not serious | not serious | serious | not serious | RR 1.05(0.99-1.11) | moderate |
| Clinical treatment success based on CE for HAP | | | | | | | | | |
| Tigecycline vs Imipenem/cilastatin | 2 | RCT | not serious | not serious | not serious | very serious | not serious | RR 0.93(0.8-1.07) | low |
| Carbapenems vs other β-lactams/FQ ± aminoglycosides | 11 | RCT | serious | serious | not serious | very serious | not serious | RR 1(0.72-1.4) | very low |
| Clinical treatment success based on CE for VAP | | | | | | | | | |
| Monotherapy vs combination therapy | 2 | RCT | not serious | serious | not serious | very serious | not serious | OR 1.02(0.21-4.95) | very low |
| Carbapenems vs non-carbapenem | 3 | RCT | serious | not serious | not serious | serious | not serious | RR 1.22(0.99-1.34) | moderate |

CAP: community-acquired pneumonia; HAP：hospital-acquired pneumonia; VAP: ventilator-associated pneumonia; NP: nosocomial pneumonia; RCT: randomized controlled trials; CE: clinically evaluable; RR: risk ratios; FQ: fluoroquinolones; M: macrolide; Q: quinolones.

**References**

1. Yuan X, Liang B-B, Wang R, Liu Y-N, Sun C-G, Cai Y, et al. Treatment of community-acquired pneumonia with moxifloxacin: a meta-analysis of randomized controlled trials*.* Journal of chemotherapy (2012) 24(5):257-67.

2. Vardakas KZ, Siempos II, Grammatikos A, Athanassa Z, Korbila IP, Falagas ME. Respiratory fluoroquinolones for the treatment of community-acquired pneumonia: a meta-analysis of randomized controlled trials*.* CMAJ : Canadian Medical Association journal (2008) 179:1269.

3. Liu S, Tong X, Ma Y, Wang D, Huang J, Zhang L, et al. Respiratory Fluoroquinolones Monotherapy vs. β-Lactams With or Without Macrolides for Hospitalized Community-Acquired Pneumonia Patients: A Meta-Analysis*.* Front Pharmacol (2019) 8:10:489.

4. Skalsky K, Yahav D, Lador A, Eliakim-Raz N, Leibovici L, Paul M. Macrolides vs. quinolones for community-acquired pneumonia: meta-analysis of randomized controlled trials*.* Clinical Microbiology and Infection (2013) 19:370-8.

5. Raz-Pasteur A, Shasha D, Paul M. Fluoroquinolones or macrolides alone versus combined with β-lactams for adults with community-acquired pneumonia: Systematic review and meta-analysis*.* International Journal of Antimicrobial Agents (2015) 46:242-8.

6. Salkind AR, Cuddy PG, Foxworth JW. Fluoroquinolone Treatment of Community-Acquired Pneumonia: A Meta-Analysis*.* Annals of Pharmacotherapy (2002) 36:1938-1943.

7. Robenshtok E, Shefet D, Gafter-Gvili A, Paul M, Leibovici L. Empiric antibiotic coverage of atypical pathogens for community-acquired pneumonia in hospitalized adults*.* Cochrane Database Syst Rev (2012) 9:CD004418.

8. Mills GD, Oehley MR, Arrol B. Effectiveness of beta lactam antibiotics compared with antibiotics active against atypical pathogens in non-severe community acquired pneumonia: meta-analysis*.* BMJ (online) (2005) 330:456.

9. Lan SH, Chang SP, Lai CC, Lu LC, Chao CM. Efficacy and Safety of Ceftaroline for the Treatment of Community-Acquired Pneumonia: A Systemic Review and Meta-Analysis of Randomized Controlled Trials*.* Journal of Clinical Medicine (2019) 8:824.

10. Horita N, Otsuka T, Haranaga S, Namkoong H, Miki M, Miyashita N, et al. Beta-lactam plus macrolides or beta-lactam alone for community-acquired pneumonia: A systematic review and meta-analysis*.* Respirology (2016) 21(7):1193-200.

11. Pakhale S, Mulpuru S, Verheij TJ, Kochen MM, Bjerre LM. Antibiotics for community-acquired pneumonia in adult outpatients*.* Cochrane database of systematic reviews (Online) (2014) 10:CD002109.

12. Wen J, Chen F, Zhao M, Wang X. Solithromycin monotherapy for treatment of community-acquired bacterial pneumonia: A meta-analysis of randomised controlled trials*.* International Journal of Clinical Practice (2019) 73(5):e13333.

13. Cai Y, Wang R, Liang B, Bai N, Liu Y. Systematic review and meta-analysis of the effectiveness and safety of tigecycline for treatment of infectious disease*.* Antimicrobial Agents & Chemotherapy (2011) 55:1162-72.

14. Lei Z, Rui W, Matthew FE, Liang-An C, You-Ning L. Gemifloxacin for the treatment of community-acquired pneumonia and acute exacerbation of chronic bronchitis: a meta-analysis of randomized controlled trials*.* Chin Med J (Engl) (2012) 125:687-695.

15. Shen F, Han Q, Xie D, Fang M, Zeng H, Deng Y. Efficacy and safety of tigecycline for the treatment of severe infectious diseases: an updated meta-analysis of RCTs*.* International Journal of Infectious Diseases Ijid Official Publication of the International Society for Infectious Diseases (2015) 39:25-33.

16. Chen CK, Cheng IL, Chen YH, Lai CC. Efficacy and Safety of Sitafloxacin in the Treatment of Acute Bacterial Infection: A Meta-analysis of Randomized Controlled Trials*.* Antibiotics (2020) 9:106.

17. Zhang Y, Wang Y, Driel MLV, McGuire TM, Zhang T, Dong Y, et al. Network meta-analysis and pharmacoeconomic evaluation of antibiotics for the treatment of patients infected with complicated skin and soft structure infection and hospital-acquired or ventilator-associated penumonia*.* Antimicrob Resist Infect Control (2019) 8:72.

18. Jiang H, Tang RN, Wang J. Linezolid versus vancomycin or teicoplanin for nosocomial pneumonia: meta-analysis of randomised controlled trials*.* Eur J Clin Microbiol Infect Dis (2013) 32:1121-1128.

19. Qu XY, Hu TT, Zhou W. A meta-analysis of efficacy and safety of doripenem for treating bacterial infections*.* The Brazilian Journal of Infectious Diseases (2015) 19(2):156-62.

20. Shorr AF, Susla GB, Kollef MH. Quinolones for treatment of nosocomial pneumonia: a meta-analysis*.* Clinical Infectious Diseases An Official Publication of the Infectious Diseases Society of America (2005) 40:Suppl 2:S115-22.

21. Nicholas O"Donnell J, Rhodes NJ, Lopez J, Jett R, Scheetz MH. Carbapenems versus alternative β-lactams for the treatment of nosocomial pneumonia: A systematic review and meta-analysis*.* Int J Antimicrob Agents (2018) 52(4):451-458.

22. Siempos II, Vardakas KZ, Manta KG, Falagas ME. Carbapenems for the treatment of immunocompetent adult patients with nosocomial pneumonia*.* European Respiratory Journal (2007) 29:548.

23. Arthur LE, Kizor RS, Selim AG, Driel MLV, Seoane L. Antibiotics for ventilator-associated pneumonia*.* Cochrane Database Syst Rev (2016) 10(10):CD004267.

24. Sweeney DA, Kalil AC. Why don't we have more inhaled antibiotics to treat ventilator-associated pneumonia? Clinical Microbiology and Infection (2019) 25:1195-1199.

25. Kalil AC, Metersky ML, Michael K, John M, Sweeney DA, Palmer LB, et al. Management of Adults With Hospital-acquired and Ventilator-associated Pneumonia: 2016 Clinical Practice Guidelines by the Infectious Diseases Society of America and the American Thoracic Society*.* Clinical Infectious Diseases (2016) 575-582.

26. Rui L, Fu L, Rui D, Sheng-qian L, Yuan P, Mei H, et al. Efficacy and safety of aerosolized inhalation of aminoglycosides in the treatment of pulmonary infections caused by multiple/extensively-drug resistant bacteria: A systematic review and Meta-analysis*.* Chin J Antibio (2019) 44.

27. Liu Y, Li J, Liao L, Yu H, Zhang B. Clinical efficacy of moxifloxacin in the treatment of Mycoplasma pneumonia*.* Chin J Infect Chemother (2010) 10:349-53.

28. Garin N, Genné D, Carballo S, Chuard C, Eich G, Hugli O, et al. β-Lactam monotherapy vs β-lactam-macrolide combination treatment in moderately severe community-acquired pneumonia: a randomized noninferiority trial*.* Jama Internal Medicine (2014) 174:1894-901.
